# The Street Folk
**London Labour and the London Poor:* a Cyclopædia of the Condition and Earnings of those that *will* work, those that *cannot* work, and those that *will not* work. By Henry Mayhew. Vols. i. ii. and iii. 8vo. London: Griffin, Bohn, & Co. 1861.—*Ragged London in* 1861. By John Hollingshead. 8vo. London: Smith, Elder, and Co. 1861.


**Published:** 1862-01

**Authors:** 


### Art. VIII.—THE STREET FOLK.*

"How is this, that men of science, osteologists, and surgeons, beat some poets in respect of nature—count nought common or unclean—spend raptures upon perfect specimens of indurated veins, distorted joints, or beautiful new cases of curved spine; while we are shocked at nature's falling off, and shrink back from her warts and blains? Nay, we will not, when she sees us, even look at her to say 'God bless her!'" So questions the accomplished and lamented author of *Aurora Leigh,* and adds:—

That's our wrong;
For that, she will not trust us often with
Her larger sense of beauty and desire,
But tethers us to a lily or a rose,
And bids us diet on the dew inside,
*Left ignorant that the hungry beggar boy*
(*Who starves unseen against our abstract eyes,*
*And wonders at the gods that we must be,*
*To pass so careless for the oranges!*)
Bears yet a breastful of a fellow-world,
To this world, undisparaged, undespoiled,
And (while we scorn him for a flower or two,
As being, Heaven help us, less poetical,)
Contains himself, both flowers and firmaments,
And surging seas and aspectable stars,
And all that we would push him out of sight
In order to see nearer.

Well may the poet exclaim—

Let us pray
God's grace to keep God's image in repute!

To all those who would utter the poet's aspiration, and would learn that "larger sense of beauty" of which she so eloquently descants, we would commend Mr. Mayhew's great work (thanks to the opportunity afforded by its re-issue and promised completion). The three volumes already published, and which have been several years before the public, refer to that class of the population of which the "hungry beggar boy" is the poet's type—the *street folk.* They teach us, chiefly from the mouth of the people themselves, the intimate life and habits of thought of that section of the population which forms the greater bulk of what have been significantly termed the *dangerous classes*— dangerous from their vices,

* *London Labour and the London Poor:* a Cyclopædia of the Condition and Earnings of those that *will* work, those that *cannot* work, and those that *will not* work. By Henry Mayhew. Vols. i. ii. and iii. 8vo. London: Griffin, Bohn, & Co. 1861.—*Ragged London in* 1861. By John Hollingshead. 8vo. London: Smith, Elder, and Co. 1861.

their ignorance, and their misery. From them we learn how vice, even in its worst haunts, is not altogether vicious; how ignorance is not altogether brutal; and how misery is not altogether degrading. Poesy starts back astounded to find a heroic endurance and noble self-denial, of which history recounts but few such great examples, an every-day event in the loathsome back slums of our great metropolis; philanthropy blushes with shame to find itself outdone, again and again, by the wretched people it seeks to succour; and religion even not seldom receives a lesson from the patient virtue of the irreligious. It has often been said, and with no small truth, that to determine the most efficient means of raising the mental and moral standard of the lower and more degraded classes of a people is the great problem of the age. It is in clearly pointing out the first requirement for the solution of this problem, and working it out in regard to a town population, that the peculiar value of Mr. Mayhew's work consists. Only those who know personally the class of people to which the work is devoted, can truly appreciate the gigantic task which Mr. Mayhew imposed upon himself, the rare and indomitable industry which must have been exerted in collecting his materials, and the acuteness and ability manifested in the use of them; but none can read his volumes without failing to admire the excelling literary talent with which he has spread out his stores before the public—a talent, the charm of which might tempt a reader through his encyclopædic work, apart from almost any other consideration.

But some readers may object that Mr. Mayhew's work is now getting comparatively old, and, in some respects, out of date. Alas! neither the subject nor the work can become old in this generation. For many years to come, it will be the text-book of our street folk; and in all times the most valuable work on their intimate social history in the present day. The evils which have given rise to and which foster this class, and the class itself, are the slow growth of ages; the efforts which have been specifically directed towards its amelioration are but the work of a day. Hence it is that we have taken advantage of the republication of the book thus once more to direct especial attention to it. If, however, any doubt should rest upon the mind of the reader, let him take up Mr. Hollingshead's work on *Ragged London in* 1861, and he may there readily satisfy himself that the London of to-day is in all essential respects the London of ten years ago. Neither he nor we would underrate the great philanthropic efforts directed to the mental, moral, and physical improvement of the street folk in the interval; but the magnitude of the evil is too commonly disregarded, from its being hid, as it were, in the foul byways and alleys which lurk behind the great thoroughfares, east, west, north, and south, and, as a consequence, the influence of the efforts referred to is overrated.

Mr. Hollingshead's work consists chiefly of a series of letters published in the *Morning Post,* in January, 1861, under the title of "London Horrors," and which were occasioned by the unexampled severity of the winter, and "an amount of national distress not at all exceptional in the cold season." Mr. Hollingshead visited the impoverished districts in every part of the metropolis, and ascertained by personal inspection their actual condition, as well as that of the population, gathering also trustworthy information concerning the charitable resources of each district and their administration. Familiar with the nooks and corners of London for many years, and a practised observer, the results of Mr. Hollingshead's investigation possess far more than a transitory interest, and these being conveyed to the reader with all the facility and vigour of an accomplished writer, his book is of unusual interest.

Will our readers pardon a hint *apropos* of the present period of the year? Few things probably contribute more to impede the satisfactory working of efforts for the amelioration of the condition of the lower classes in towns, than the want of precise knowledge of their actual state among a vast body of the middle classes, and consequently of true and active interest in the subject. Now, it is a pleasant custom at Christmas and the New Year to commemorate the time by gifts among friends. Let those who are interested in all questions concerning the weal and woe of the nether classes of our population, give to their gifts a highly practical character, and yet one fully befitting the time-honoured charity and good-will of the season by presenting Mayhew's and Hollingshead's works. It might be that a glance at these books, when the heart is overflowing with home thoughts and joys, would divert the mind into a track which would lead ultimately to the hearths of, and prove the path of relief to, many a wretched home, as well as the way of escape to many of the lost and deserted.

The *street folk* afford a study of remarkable psychological and physiological interest, and Mr. Mayhew has happily sketched the many points of comparison which exist between them and nomadic tribes. "The nomad," he says, "is distinguished from the civilized man by his repugnance to regular and continuous labour; by his want of providence in laying up a store for the future; by his inability to perceive consequences ever so slightly removed from immediate apprehension; by his passion for stupifying herbs and roots, and when possible, for intoxicating fermented liquors; by his extraordinary powers of enduring privation; by his comparative insensibility to pain; by an immoderate love of gaming, frequently risking his own personal liberty upon a single cast; by his love of libidinous dances; by the pleasure he experiences in witnessing the suffering of sentient creatures; by his delight in warfare and all perilous sports; by his desire for vengeance; by the lowness of his notions as to property; by the absence of chastity among his women, and his disregard of female honour; and lastly, by his vague sense of religion, his rude idea of a Creator, and utter absence of all appreciation of the mercy of the Divine Spirit." The nomadic races of England include the vagrants, pickpockets, prostitutes, street-sellers (a large and important class, the chief agents in retailing food to the poor), street-performers, cabmen, coachmen, watermen, &c. The life of these classes partakes more or less of the vagabond, and in all there is noticed a greater development of the animal than of the intellectual and moral nature of man; they all also (like the nomadic tribes proper) possess more or less a peculiar physical conformation, use a slang language, and are distinguished for their lax ideas of property, their general improvidence, their repugnance to continuous labour, their disregard of female honour, their love of cruelty, their pugnacity, and their utter want of religion.

Happily there are many points of relief in this character, points which afford a good ground of hope to the philanthropist. The street folk are exceedingly susceptible to kindness, and often among themselves display rare traits of honesty and self-denial. Mrs. Chisholm lent out at different times among them no less than 160,000*l.*, and *the whole of this large sum was returned with the exception of* 12*l.*! Mr. Hollingshead tells of a woman, who works at clump-box making, picking up an orphan boy from the streets and giving him a place amongst her own children. He rightly adds, "Many cases of such self-sacrifice and large-hearted generosity may be easily found amongst the poor. The cases of heroic endurance under the most frightful trials are even more frequent, and they make us respect these creatures even in their dirt and rags." Mr. Mayhew's work abounds in illustrations of this truth; and Mr. Hollingshead gives several singularly interesting and suggestive examples of the manner in which virtue may appear even among the most wretched and miserable of our population. Writing of the female population found at the back of Whitechapel, he says, "The best-paid occupation appears to be prostitution, and it is a melancholy fact that a nest of bad houses in Angel Alley, supported chiefly by the farmers' men who bring hay and straw to Whitechapel market twice a-week, are the cleanest-looking dwellings in the district. The windows have tolerably neat green blinds, the doors have brass plates, and inside the houses there is comparative comfort, if not plenty. While the wretched virtuous population are starving in black holes, or creeping out in the hour of their wildest prosperity to purchase sixpennyworth of refuse meat from the stall opposite the greasy, sawdusty shambles, the inhabitants of this court of vice know little, at least for a few years, of want and suffering." (p. 49.) And again, writing of St. George's-in-the-East, he says, "It is a curious social fact, that when a particular street reaches a certain depth of poverty, it becomes purified from its vice; for though thieves and prostitutes do not become converted in a period of scarcity, they inevitably shift their quarters." (p. 62.) Writing of Agar Town, he also remarks, "No known thieves or prostitutes are found in the neighbourhood; its lowest part is too poor and miserable for that; and what vices it has arise largely from dirt and overcrowding." (p. 137.)

We do not propose to discuss systematically, or at any length, the characteristics of the *street folk*, but rather to illustrate one or two important questions bearing upon the fostering and perpetuating causes of those characteristics.

And, first, of the dwellings of the street folk. We are perhaps a little too apt to imagine, from the lavish attention which has of late years been devoted to the dwellings of the poor in towns, that, even if these have not been so widely improved as might have been wished, still their downward course was, in a measure, prevented. Mr. Hollingshead rudely dispels the pleasing illusion. He tells us, writing of Bethnal Green, Shoreditch, and Spitalfields, "I have known the neighbourhood I am describing for twenty years, and, if anything, it seems to me to be getting dirtier and more miserable every year. Old houses, in some few places, have been taken away, simply because they fell to pieces; but the new houses erected within the last ten years show little advance in the art of building dwellings for the poor. The whole present plan and arrangement of the district is against improvement, and the new structures sink to the level of the old." (p. 72.) And again, writing of many streets within the City borders, he says, "The blind alleys in coal-mines, the slimy passages of district sewers, anything that is dark and filthy, may be compared to these places. I have known them for years, and whatever sanguine clergymen or apologetic inspectors may say to the contrary, they get worse every day, and can never be improved. The tinkering of a drain, the whitewashing of a ceiling, will never remove the evils of their original structure." He adds, "They are filled with labourers, artisans, needlewomen, and girls employed in many fancy trades; and the capital and enterprise of the City of London are largely responsible for them all." (p. 18.)

This *moral responsibility* of the wealthier classes for much of that which is most revolting and painful among the poorer classes, is far too little considered or understood in discussing the physical and mental state of the latter. No influence contributes more powerfully in perpetuating and confirming the worst vices of the street-folk and the poorer classes generally; none so greatly impedes the success of the efforts made for the amelioration and improvement of their condition as the apathy, neglect, or positive evil example of the wealthier classes. Speaking of streets in Marylebone and its outskirts, which are notorious haunts of thieves and prostitutes, and in which the lowest threepenny lodging-houses abound, Mr. Hollingshead says:—"In this case, the blot upon the district is not made more foul by being the property of a parish officer. Rate collectors, registrars, and active vestrymen are too often the proprietors of these places, making an extra profit by their local knowledge. Sometimes the land such pest-houses stand upon is Crown or Church property, and the rent, and more, has to be expended in the work of counteracting their influence." (p. 148.)

Mr. Mayhew tells of nearly a dozen brothels in Westminster, "owned by a *member of a strict Baptist Church, and the son of a deceased minister*," and who "lives in great splendour in one of the fashionable streets in Pimlico" (vol. I. p. 317). Mr. Godwin speaks of houses in a court in Clerkenwell, so dilapidated that few would suppose they were inhabited, yet the rooms let at exorbitant rents, and in one of the houses two of the rooms accommodated at night twenty-five persons. The houses belong to a gentleman at Notting-hill, by whom they are let to a chimney-sweeper, who lives on the spot and sub-lets them.*

Again, in further illustration of this question, a costermonger remarked to Mr. Mayhew, when reproved by him for dishonest trickery:—

"It aint *we* as makes coffee out of sham chickory; it aint *we* as makes cigars out of rhubarb leaves; *we* don't make duffer's handkerchiefs, nor weave cotton things and call them silk. If we quacks a bit, does *we* make fortins by it as shopkeepers does with their ointment and pills? If we give slang weights, how many rich shopkeepers is fined for that there? And how many's never found out? And when one on 'em's fined, why he calculates how much he's into pocket, between what he's made by slanging and what he's been fined, and on he goes again. *He* didn't know that there ever was short weight given in his shop, not *he!* no more do *we* at our stalls or barrows! Who 'dulterates the beer? Who makes old tea-leaves into new? Who grinds rice among pepper? And as for smuggling—but nobody thinks there's any harm in buying smuggled things. What *we* do is like that pencil you're writing with to a great tree, compared to what the rich people does. O, don't tell me, sir, a gentleman like you, that sees so much of what's a going on, must know *we're* better than the shopkeepers are."†

---

* *London Shadows*, p. 12. † Mayhew, vol. i. p. 474.

These facts speak for themselves, and others might be quoted in abundance indicative of other and even more powerful influences for evil among the street folk. "*That's none of my business,*" said a dust contractor when spoken to upon the morals of his workmen; "*they do my work, and that's all I want with them, and all I care about. . . . Let every one look out for themselves as I do, and then they need not care for anyone.*" This sentiment is by no means an unfamiliar one in the trading world, and, indeed, influences widely the business transactions of those who it might be thought, would escape from the *grinding* habits grafted too generally upon ordinary trade pursuits. A correspondent of Mr. Mayhew's, writing of the *contract* system, says :—

"Depend upon it, sir, the father of wickedness himself could not devise a more malevolent or dishonest course than that now very generally pursued by those who should be, of all others, the friends of the poor and working man. The Government and the great West-end clubs have carried their transactions to such a low level in this respect that it seems to be the only question with them, who will work lowest or supply goods at the lowest figure? And this, too, totally irrespective of the circumstance whether it may not reduce wages or bankrupt the contractor. No matter whether a party who has executed the work required for years, be noted for paying a fair and remunerating price to his workmen or sub-tradesmen, and bears the character of a responsible and trustworthy man—all this is nothing; for somebody, who may be, for aught that is cared, deficient in all these points, will do what is needful at *so much* less; and then, unless willing to reduce the wage of his workpeople, the long-employed tradesman has but the alternative of losing his business or cheating his creditors. And then, to give a smack to the whole affair, the 'Stationery Office' of the Government, or the committee of the club, will congratulate themselves and their auditors on the fact that a diminution in expenses has been effected; a result commemorated perhaps by an addition of salary to the officials in the former case, and of a 'cordial vote of thanks' in the latter. I do not write 'without book.' I can assure you on these matters, for I have long and earnestly watched the subject, and could fill many a page with details."*

The lesson is obvious. Much that is most revolting and hideous in the physical, mental, and moral condition of the impoverished classes is entirely dependent upon influences external to them, and over which they have no control. Hence good from any efforts made for the improvement and amelioration of the condition of these classes, must of necessity be in a great measure limited by the nature and results of efforts made for the removal of the causes of this condition existing among other classes. Yet it may, we think, be safely asserted that the efforts

* Vol. ii. p. 331.

made for this latter end, are by no means comparable, nay, indeed, that they are far inferior, both in energy and precision, to those made to command the former.

A street-life is the last resort of the impoverished labourer or artisan to "*pick up a crust*" (as he emphatically words it); and *crossing-sweeping* is the last resort of the street folk to earn one. Among the street folk we find the struggle for existence in its sternest and severest form, and it is the intensity of the struggle which is the chief agent in developing the mental and moral, as well as physical characteristics of the class. Among the costermongers, the vagrants, and the criminal class, there are a limited number to whom a street life is hereditary, and a few resort to it from a mere vagabond feeling—a restless aversion to the steady and systematic habits of more profitable, but not more laborious labour. But even the best among the street folk barely keep themselves at all times above the level of physical comfort, few reach the happy medium of persistent comfort, and with the vast majority life is a stern protracted struggle with actual privation. Two or three days' continued rain plunges many thousand costermongers and street-artisans into actual want; and the frost of last winter told a terrible tale of the dependency of these people on their day's receipts, whatever these might be, for the day's food. "No one," said a costermonger, "but those actually connected with the streets can tell the exertion, anxiety, and difficulties we have to undergo; and I know for a fact it induces a great many to drink that would not do so, only to give them a stimulant to bear up against the troubles that they have to contend with; and so it ultimately becomes habitual." "We don't live, we starruve," said an Irishwoman, an orange-hawker; "we git a few 'taties, and sometimes a plaice. To-day I've not taken 3d. as yit, sir, and it's past three. Oh, no, indeed and indeed, thin, I don't make 9d. a day. We live accordingly, for there's 1s. 3d. a week rint." "Lives!" exclaimed a bootlace woman, "starves you mean; for all yesterday I only took a farthing. But," she added, "anything's better than the house (workhouse). I'll live on 4d. a day, and pay rent and all, and starve half my time, rather nor the great house." "I live on brid, and 'taties and salt," said another woman, "and a herrin' sometimes. I niver taste beer, and not often tay, but I sit here all day, and I feel the hunger this day and that day. It goes off though, and I have nothin' to ate. I don't know why, but I wont deny the goodness of God, to bring such a thing about. I have lived for a day on a pinny, sir: a ha'pinny for brid, and a ha'pinny for a herrin', or two herrin's for a ha'penny and 'taties for the place of brid. I've changed apples for a herrin' with a poor man, God rewarrud him.

Sometimes I make on to 6d. a day, and sometimes I *have* made 1s. 6d., but I think that I don't make 5d. a day—arrah, no, thin, sir! one day with the other, and I don't work on Sunday, not often. If I've no mate to ate, I'd rather rist. I never miss mass on a Sunday. A lady gives me a rag sometimes, but the bitter time's comin'. If I was sick I don't know what I'd do, but I would sind for the praste, and he'd counsil me. I could read a little onst, but I can't now."

"Many a one goes down to the market," said a watercress girl, "with only three half-pence, and glad to have that to get a half-penny, or anything, so as to earn a mouthful of bread—a bellyful, that they can't get no how. At many a time I walked through the streets, and picked a piece of bread that the servants chucked out of the door, may be to the birds; I've gone and picked it up when I've been right hungry. Thinks I, I can eat that as well as the birds." "I don't have no dinner," said a watercress girl, eight years old; "mother gives me two slices of bread-and-butter and a cup of tea for breakfast, and then I go till tea and has the same. We has meat of a Sunday, and of course, I should like to have it every day. Mother has just the same to eat as we has, but she takes more tea, three cups, sometimes." The same little thing also said, of winter, "I gets up in the dark by the light of the lamp in the court. When the snow is on the ground, there's no creases. I bears the cold—you must, so I puts my hands under my shawl, though it hurts 'em to take hold of the creases, especially when we takes 'em to the pump to wash 'em. No; I never see any children crying—it's no use." "On a wet day," said a crippled street-seller, "When I can't get out, I often go without food. I may have a bit of bread-and-butter given me, but that's all—then I lie a-bed. I feel miserable enough when I see the rain come down of a week day, I can tell you. Ah! it *is* very miserable indeed lying in bed all day, and in a lonely room, without perhaps a person to come near one—helpless as I am—and hear the rain beat against the windows, and all that without nothing to put in your lips." "No; I've earned no money to-day," said another man: "I have had a piece of dried bread that I steeped in water to eat. I haven't eat anything else to-day; but pray, sir, don't tell anybody of it. I could never bear the thought of going into the great house; I'm so used to the air, that I'd sooner die in the streets, as many I know have done. I have known some of our people, who have set down in the street with their basket alongside them, and died." In the *Morning Chronicle* of November last, was the following paragraph:—

"On Saturday morning, a man of about forty, was found huddled up and dead in an outbuilding near the Mint, in Southwark; and another about forty, in the New-road, Marylebone, both having perished from want and exposure to the inclemency of the weather."

"Why, I stood at this bench, with my child, only ten years of age," said the wife of a fancy-cabinet maker, who worked also with her children in the same business, "from four o'clock on Friday morning till ten minutes past seven in the evening, without a bit to eat or drink. I never sat down a minute from the time I began till I finished my work, and then I went out to sell what I had done. I walked all the way from here (Shoreditch) down to the Lowther Arcade, to get rid of the articles." Here she burst out into a violent flood of tears, saying, "Oh! sir, it is hard to be obliged to labour from morning to night as we do, all of us, little ones and all, and yet not to be able to live by it either." "After eight hours," said a coster-girl, carrying a tray strapped to her loins (to turn for a moment to the labour of the streets) "after eight hours, it swaggers me like drink." "The life is such a hard one," said a coster, "that a girl is ready to get rid of a *little* of the labour at any price; that she readily enters into concubinage with the first coster-lad who takes a 'fancy' to her." A girl, however, "seldom takes up with a lad before she is sixteen, though some of them, when barely fifteen, or even fourteen, will pair off."

"I'm often very badly off, indeed, very badly," said a street fish-seller; "and the misery of being hard-up, sir, is not when you're making a struggle to get out of your trouble; no, nor to raise a meal off herrings, that you've given away once, but when your wife and you's sitting by a grate without a fire, and putting the candle out to save it, a-planning how to raise money. 'Can we borrow that?' 'Can we manage to sell if we can borrow? Shall we get from very bad to the parish?' Then, perhaps, there is a day lost, and without a bite in our mouths, trying to borrow, let alone a little drop to give a body courage, which perhaps is the only good use of spirits after all. That's the pinch, sir. *When the rain you hear outside puts you in mind of drownding!*"

Add to these facts, the knowledge that the diet of the street-folk when at the best, consists chiefly of fish ("sprats *is* a blessing to the poor, fresh herrings is a blessing too," said a street-seller), and that their poverty as well as that of their neighbours, is such that it is credibly asserted of one district, St. Paul's, Marylebone, that one-half of the parishioners, about 5000 people, have nearly all their garments in pawn, and it needs no scientific disquisition to show the necessary moral and intellectual degeneration of the people so circumstanced, and in what fashions this will show itself. The marvel is, that when hunger so often prompts to theft, so few comparatively should thieve; when prostitution offers a ready opening for some relief, chastity should still be found; where godliness is almost unknown, many virtues should yet abound; where the "*move on*" of the policeman is too often a bar to earning a bare subsistence, the law should still be respected. "To show the temptations which beset the poor," Mr. Mayhew writes elsewhere,*

"I give the statement of a woman known to all her neighbours as a very thrifty housewife, and an active, industrious woman. Her children's, her own, and her husband's clothing, scant and old as it was, all showed great care-taking; and her home was, at the least, tidy. She was the wife of one of the labouring men engaged in discharging the timber-ships, and who are called 'lumpers,' from the fact of their being employed by some publican who contracts for the job 'in a lump.' This system is fraught with every kind of evil to the workmen and their families, the men being forced, or rather 'expected,' to spend the greater portion of their earnings in drink at the house of their employer. The ballast-heavers suffer from the same system. The houses of such people hardly deserve the name of lairs. A few years back, a little after Christmas, the 'lumper's' wife and her husband had been out all day, penniless, seeking for work, and had returned to their room, a little before dusk, without having earned a farthing, the wife then suckling her first child, which was two months old. She felt faint from long want of nourishment, and the only thing in the house on which she thought it possible to raise a penny was a glass tumbler. 'That very tumbler,' she said, 'which you see on the table; everything but that had gone to the pawn-shop. Look, it cost 5½d., and I went and tried to sell it for 2d. I couldn't sell it at all, as the dealer had too many of such things. I then went to a neighbour, and said, 'Mrs. B——, for God's sake lend me 2d. on this glass, for we're starving.' 'Mrs. ——,' said she, 'I'm sure you should have 3d.; but I haven't 3d. nor a halfpenny.' Well, when I got back it was dark; my husband had gone to bed, such as it was (for we had neither blankets nor sheets left to cover us), as the best way to forget he was hungry and cold. We hadn't a bit of fire nor candle, but a light came, from a lamp in the street, through the window. I sat down by the fire, that wasn't in, to suckle my child (poor little Bill! he's a fine lad, now), and I found I had hardly any milk. It seemed to me the child must be starved, since I had nothing to give it. All at once a thought came into my head, and I said to myself, 'Yes, I'll cut my own throat and little Bill's, too.' I determined I would. Then I said to myself, 'No, I won't; for if I can cut my own throat, I know I can't cut the child's; so it'll be little use. I'll go to the Water-works, and jump in, with him in my arms.' I got up to do it; and then another thought darted into my mind, and I laid down the child on the chair, and rushing up to my husband, shook him violently, as I cried, 'You villain, I'll cut your throat, I will!' He jumped up and

---

* *Meliora*, edited by Visct. Ingestre, p. 274. 1852.

seized hold of me, and then I felt how bad I'd been. But one's passion must have some vent, so I seized by the spout that very kettle you see there, and I smashed it on the floor. It was the first thing that came to hand. After that I felt calmed a bit, and began to see how wicked I'd been; and then I fell down on my knees, and cried like a child, for I was thankful to God I'd been preserved. I soon went to bed, and there I prayed never to feel the like again!' This statement was made with perfect simplicity; it came out incidentally, and the poor woman had no reason to believe that it would be printed."

Let us, while condemning the vices and seeking to remedy the evils which exist among the most impoverished, not forget the virtues and good which they so largely manifest. The latter constitute our great hope for the future of the *street-folk* and their fellows; and they who would learn accurately to appreciate what is good and what is evil, and the sources of both the good and the evil,—learn, in fact, all that relates to the physical, moral, and intellectual state of these people, should turn to Mr. Mayhew's pages.

---

